# Crystal structure of 2-benzyl­amino-4-(4-bromo­phen­yl)-6,7-di­hydro-5*H*-cyclo­penta­[*b*]pyridine-3-carbo­nitrile

**DOI:** 10.1107/S2056989015002820

**Published:** 2015-02-21

**Authors:** R. A. Nagalakshmi, J. Suresh, S. Maharani, R. Ranjith Kumar, P. L. Nilantha Lakshman

**Affiliations:** a Department of Physics, The Madura College, Madurai 625 011, India; b Department of Organic Chemistry, School of Chemistry, Madurai Kamaraj University, Madurai 625 021, India; c Department of Food Science and Technology, University of Ruhuna, Mapalana, Kamburupitiya 81100, Sri Lanka

**Keywords:** crystal structure, cyclo­pentane ring, envelope conformation, N—H⋯N hydrogen bonding, π–π inter­actions

## Abstract

In the title compound C_22_H_18_BrN_3_, the cyclo­pentane ring adopts an envelope conformation with the central methyl­ene C atom as the flap. The dihedral angles between the central pyridine ring and the pendant benzyl and and bromo­benzene rings are 82.65 (1) and 47.23 (1)°, respectively. In the crystal, inversion dimers linked by pairs of N—H⋯N_n_ (n = nitrile) hydrogen bonds generate *R*_2_^2^(12) loops. These dimers are linked by weak π–π inter­actions [centroid–centroid distance = 3.7713 (14) Å] into a layered structure.

## Abstract 2

The packing of the title compound features N—H⋯N hydrogen bonds, which form inversion dimers, and weak aromatic π–π stacking inter­actions.

## Chemical context   

Cyano­pyridine derivatives exhibit useful anti­cancer and anti­viral activities (Cocco *et al.*, 2005 ▸; El-Hawash & Abdel Wahab, 2006 ▸). 3-Cyano­pyridine derivatives have been reported for their wide range of applications such as in their anti­microbial, analgesic, anti-hyperglycemic, anti­proliferative and anti­tumor activities (Brandt *et al.*, 2010 ▸; El-Sayed *et al.*, 2011 ▸; Ji *et al.*, 2007 ▸). As part of our ongoing work in this area, we synthesized the title compound, which contains a pyridine 3-carbo­nitrile group, and we report herein on its crystal structure. 
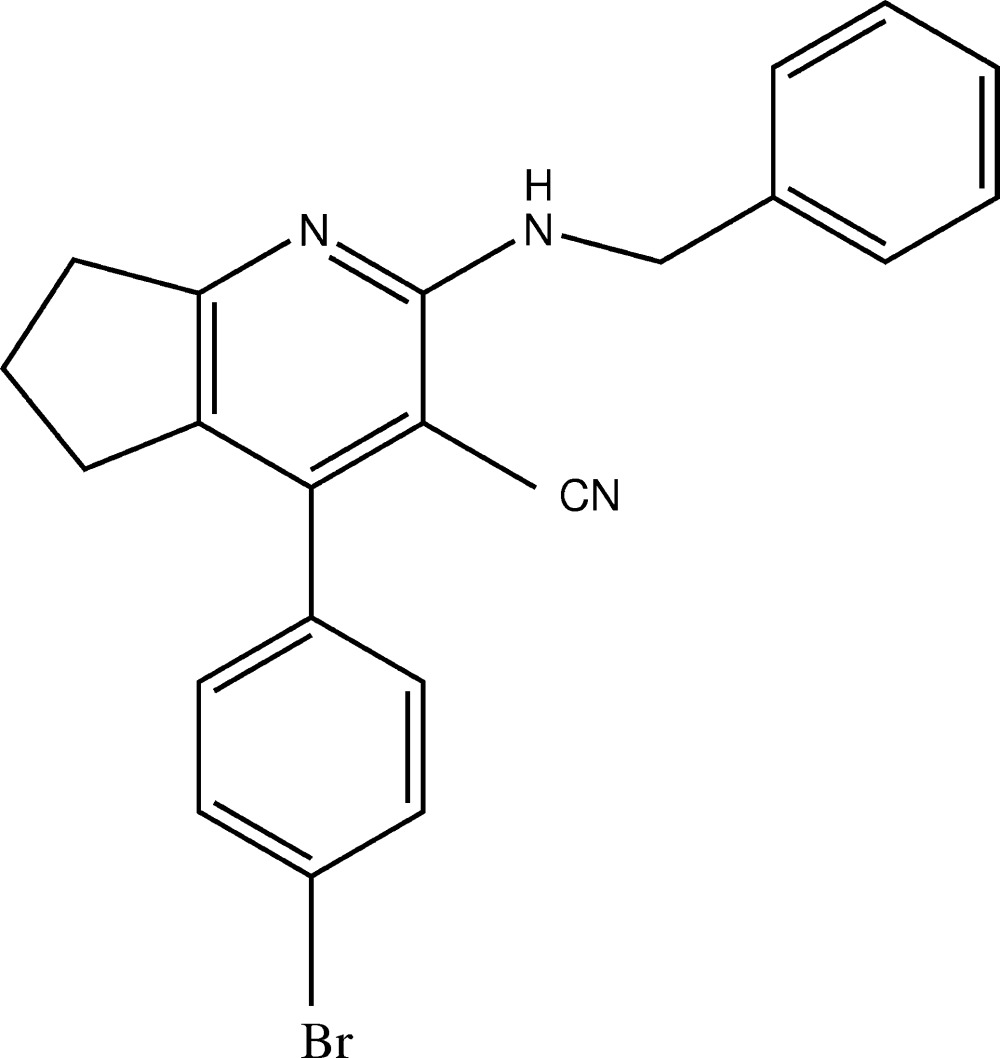


## Structural commentary   

**Figure 1:**
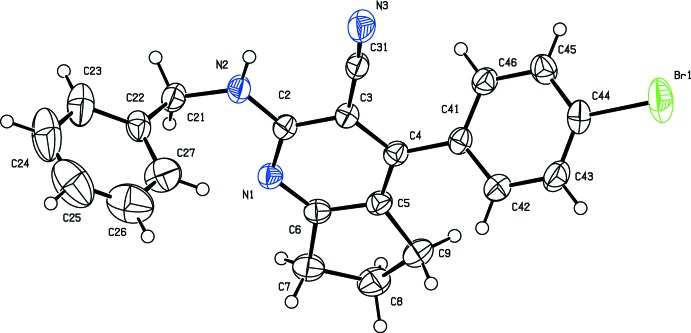
The mol­ecular structure of the title compound, with displacement ellipsoids drawn at the 30% probability level.

The mol­ecular structure of the title compound (I) is shown in Fig. 1 ▸. The nitrile atoms C31 and N3 are displaced from the mean plane of the pyridine ring by 0.1016 (1) and 0.1997 (1) Å, respectively. The cyclo­pentane ring fused with the pyridine ring adopts an envelope conformation with atom C8 as the flap, deviating by 0.3771 (1) Å from the mean plane defined by the other atoms (C5/C6/C7/C9). The amino group is nearly coplanar with the pyridine ring as indicated by the torsion angle N2—C2—C3—C4 = −178.0 (16)°. Steric hindrance rotates the benzene ring (C22–C27) out of the plane of the central pyridine ring by 82.65 (1)°. This twist may be due to the non-bonded inter­actions between one of the *ortho* H atoms of the benzene ring and atom H21*B* of the CH_2_–NH_2_ chain.

## Supra­molecular features   

**Figure 2:**
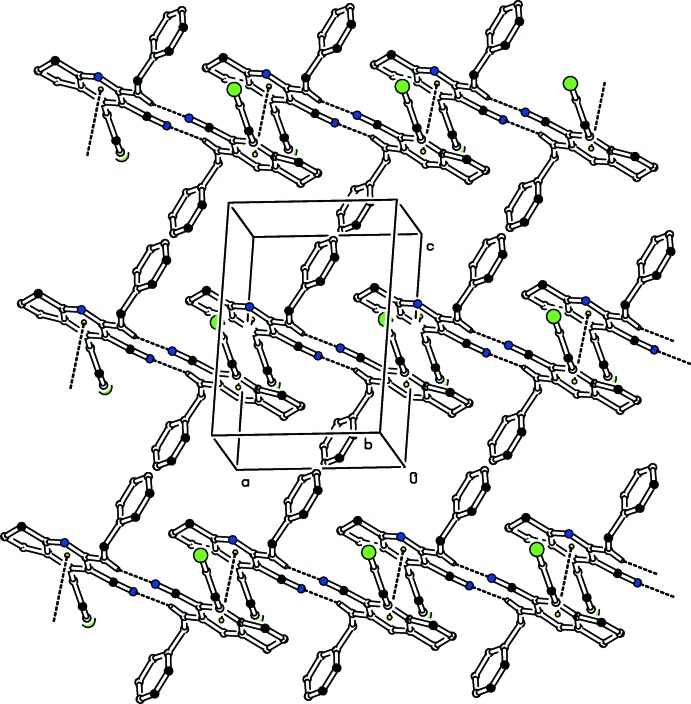
Partial packing diagram of compound (I). For clarity, H atoms bound to atoms not involved in hydrogen bonding are not shown.

In the crystal, mol­ecules are linked *via* pairs of N—H⋯N_n_ (n = nitrile) hydrogen bonds, forming inversion dimers which enclose 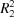
(12) ring motifs (Table 1 ▸ and Fig. 2 ▸). The dimers are further connected by slipped parallel π–π stacking inter­actions involving the pyridine rings of inversion-related mol­ecules [centroid–centroid separation= 3.7713 (12) Å, slippage = 1.018 Å; *Cg*1 is the centroid of the N1/C2–C6 ring; symmetry code: (i) −*x*, −*y*, 1 − *z*], as shown in Fig. 2 ▸.

## Database survey   

Similar structures reported in the literature include 2-(2-(4-chloro­phen­yl)-2-oxoeth­oxy)-6,7-di­hydro-5*H*-cyclo­penta­[*b*]pyridine-3-carbo­nitrile (Mazina *et al.*, 2005 ▸) and 2-benzylamino-4-(4-meth­oxy­phen­yl)-6,7,8,9-tetra­hydro-5*H*-cyclohepta­[*b*]pyridine-3-carbo­nitrile (Nagalakshmi *et al.*, 2014 ▸). In both compounds, the fused cyclo­pentane ring has an envelope conformation with the central methyl­ene C atom as the flap.

## Synthesis and crystallization   

A mixture of cyclo­penta­none (1 mmol) 1, 4-bromo benz­alde­hyde (1 mmol), malono­nitrile (1 mmol) and benzyl­amine were taken in ethanol (10 ml) to which *p*-TSA (1 mmol) was added. The reaction mixture was heated under reflux for 2–3 h. The reaction progress was monitored by thin layer chromatography (TLC). After completion of the reaction, the mixture was poured into crushed ice and extracted with ethyl acetate. The excess solvent was removed under vacuum and the residue was subjected to column chromatography using petroleum ether/ethyl acetate mixture (97:3 *v*/*v*) as eluent to obtain pure product The product was recrystallized from ethyl acetate, affording colourless block-like crystals (yield 68%; m.p. 474–478 K).

## Refinement   

Crystal data, data collection and structure refinement details are summarized in Table 2 ▸. The NH and C-bound H atoms were placed in calculated positions and allowed to ride on their carrier atoms: N—H = 0.86 Å, C—H = 0.93–0.97 Å, with *U*_iso_(H) = 1.5*U*_eq_(C) for methyl H atoms and = 1.2*U*_eq_(N,C) for other H atoms. The best crystal investigated was of rather poor quality and very weakly diffracting, with no usable data obtained above 49° in 2θ. Nonetheless, the structure solved readily and refined to give acceptable uncertainties on the metrical data.

## Supplementary Material

Crystal structure: contains datablock(s) global, I. DOI: 10.1107/S2056989015002820/hb7365sup1.cif

Structure factors: contains datablock(s) I. DOI: 10.1107/S2056989015002820/hb7365Isup2.hkl

Supporting information file. DOI: 10.1107/S2056989015002820/hb7365Isup3.cml

CCDC reference: 1048517

Additional supporting information:  crystallographic information; 3D view; checkCIF report

## Figures and Tables

**Table 1:** Hydrogen-bond geometry (, )

*D*H*A*	*D*H	H*A*	*D* *A*	*D*H*A*
N2H2N3^i^	0.86	2.23	2.974(4)	145

Symmetry code: (i) 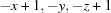.

**Table 2:** Experimental details

Crystal data	
Chemical formula	C_22_H_18_BrN_3_
*M*_r_	404.30
Crystal system, space group	Monoclinic, *P*2_1_/*c*
Temperature (K)	293
*a*, *b*, *c* ()	8.6471(3), 18.0807(5), 12.0395(4)
()	94.719(2)
*V* (^3^)	1875.94(10)
*Z*	4
Radiation type	Mo *K*
(mm^1^)	2.20
Crystal size (mm)	0.21 0.19 0.18

Data collection	
Diffractometer	Bruker Kappa APEXII
Absorption correction	Multi-scan (*SADABS*; Bruker, 2004 ▸)
*T*_min_, *T*_max_	0.967, 0.974
No. of measured, independent and observed [*I* > 2(*I*)] reflections	37065, 3084, 2232
*R*_int_	0.040
(sin /)_max_ (^1^)	0.582

Refinement	
*R*[*F* ^2^ > 2(*F* ^2^)], *wR*(*F* ^2^), *S*	0.036, 0.099, 1.05
No. of reflections	3084
No. of parameters	235
No. of restraints	1
H-atom treatment	H-atom parameters constrained
_max_, _min_ (e ^3^)	0.32, 0.54

Computer programs: *APEX2* and *SAINT* (Bruker, 2004 ▸), *SHELXS2013* (Sheldrick, 2008 ▸), *SHELXL2014* (Sheldrick, 2015 ▸) and *PLATON* (Spek, 2009 ▸).

## supplementary crystallographic information

### Crystal data

**Table:**

C_22_H_18_BrN_3_	*F*(000) = 824
*M**_r_* = 404.30	*D*_x_ = 1.432 Mg m^−^^3^
Monoclinic, *P*2_1_/*c*	Mo *K*α radiation, λ = 0.71073 Å
*a* = 8.6471 (3) Å	Cell parameters from 2000 reflections
*b* = 18.0807 (5) Å	θ = 2–31°
*c* = 12.0395 (4) Å	µ = 2.20 mm^−^^1^
β = 94.719 (2)°	*T* = 293 K
*V* = 1875.94 (10) Å^3^	Block, colourless
*Z* = 4	0.21 × 0.19 × 0.18 mm

### Data collection

**Table:**

Bruker Kappa APEXII diffractometer	2232 reflections with *I* > 2σ(*I*)
Radiation source: fine-focus sealed tube	*R*_int_ = 0.040
ω and φ scans	θ_max_ = 24.5°, θ_min_ = 2.0°
Absorption correction: multi-scan (*SADABS*; Bruker, 2004)	*h* = −10→10
*T*_min_ = 0.967, *T*_max_ = 0.974	*k* = −21→21
37065 measured reflections	*l* = −13→13
3084 independent reflections	

### Refinement

**Table:**

Refinement on *F*^2^	1 restraint
Least-squares matrix: full	Hydrogen site location: inferred from neighbouring sites
*R*[*F*^2^ > 2σ(*F*^2^)] = 0.036	H-atom parameters constrained
*wR*(*F*^2^) = 0.099	*w* = 1/[σ^2^(*F*_o_^2^) + (0.0373*P*)^2^ + 1.776*P*] where *P* = (*F*_o_^2^ + 2*F*_c_^2^)/3
*S* = 1.05	(Δ/σ)_max_ < 0.001
3084 reflections	Δρ_max_ = 0.32 e Å^−^^3^
235 parameters	Δρ_min_ = −0.54 e Å^−^^3^

### Special details

**Table:**

Geometry. All e.s.d.'s (except the e.s.d. in the dihedral angle between two l.s. planes) are estimated using the full covariance matrix. The cell e.s.d.'s are taken into account individually in the estimation of e.s.d.'s in distances, angles and torsion angles; correlations between e.s.d.'s in cell parameters are only used when they are defined by crystal symmetry. An approximate (isotropic) treatment of cell e.s.d.'s is used for estimating e.s.d.'s involving l.s. planes.

### Fractional atomic coordinates and isotropic or equivalent isotropic displacement parameters (Å^2^)

**Table:**

	*x*	*y*	*z*	*U*_iso_*/*U*_eq_	
C2	0.1424 (3)	−0.02258 (15)	0.3665 (2)	0.0368 (6)	
C3	0.1545 (3)	0.05038 (15)	0.4090 (2)	0.0370 (6)	
C4	0.0305 (3)	0.10033 (15)	0.3938 (2)	0.0373 (7)	
C5	−0.1024 (3)	0.07382 (15)	0.3339 (2)	0.0397 (7)	
C6	−0.1033 (3)	0.00227 (16)	0.2938 (2)	0.0395 (7)	
C7	−0.2548 (3)	−0.01578 (19)	0.2298 (3)	0.0538 (8)	
H7A	−0.2933	−0.0636	0.2515	0.065*	
H7B	−0.2446	−0.0158	0.1502	0.065*	
C8	−0.3606 (4)	0.0462 (2)	0.2624 (3)	0.0612 (9)	
H8A	−0.4211	0.0304	0.3225	0.073*	
H8B	−0.4312	0.0605	0.1993	0.073*	
C9	−0.2551 (4)	0.11100 (19)	0.3002 (3)	0.0566 (9)	
H9A	−0.2452	0.1457	0.2398	0.068*	
H9B	−0.2944	0.1369	0.3626	0.068*	
C21	0.2666 (4)	−0.14468 (15)	0.3414 (3)	0.0469 (8)	
H21A	0.1612	−0.1619	0.3236	0.056*	
H21B	0.3145	−0.1768	0.3989	0.056*	
C22	0.3542 (3)	−0.15117 (16)	0.2394 (3)	0.0477 (8)	
C23	0.4514 (4)	−0.2097 (2)	0.2266 (4)	0.0782 (12)	
H23	0.4681	−0.2445	0.2832	0.094*	
C24	0.5267 (6)	−0.2170 (3)	0.1270 (6)	0.1083 (18)	
H24	0.5913	−0.2572	0.1167	0.130*	
C25	0.5029 (7)	−0.1644 (4)	0.0463 (5)	0.1124 (19)	
H25	0.5518	−0.1690	−0.0193	0.135*	
C26	0.4104 (6)	−0.1059 (4)	0.0597 (4)	0.1037 (16)	
H26	0.3969	−0.0701	0.0043	0.124*	
C27	0.3361 (5)	−0.0992 (2)	0.1556 (3)	0.0746 (11)	
H27	0.2720	−0.0587	0.1642	0.090*	
C31	0.3004 (4)	0.07214 (15)	0.4625 (3)	0.0417 (7)	
C41	0.0463 (3)	0.17640 (15)	0.4382 (2)	0.0379 (7)	
C42	0.0037 (4)	0.23703 (16)	0.3719 (3)	0.0492 (8)	
H42	−0.0396	0.2292	0.2996	0.059*	
C43	0.0239 (4)	0.30833 (17)	0.4102 (3)	0.0557 (9)	
H43	−0.0034	0.3484	0.3642	0.067*	
C44	0.0850 (4)	0.31911 (16)	0.5177 (3)	0.0509 (8)	
C45	0.1276 (4)	0.26076 (16)	0.5862 (3)	0.0490 (8)	
H45	0.1689	0.2691	0.6589	0.059*	
C46	0.1085 (3)	0.18992 (16)	0.5465 (2)	0.0443 (7)	
H46	0.1377	0.1503	0.5928	0.053*	
N1	0.0134 (3)	−0.04595 (12)	0.30696 (19)	0.0404 (6)	
N2	0.2614 (3)	−0.07048 (13)	0.3849 (2)	0.0477 (6)	
H2	0.3411	−0.0554	0.4262	0.057*	
N3	0.4210 (3)	0.08573 (15)	0.5025 (3)	0.0621 (8)	
Br1	0.11061 (6)	0.41678 (2)	0.57329 (4)	0.0879 (2)	

### Atomic displacement parameters (Å^2^)

**Table:**

	*U*^11^	*U*^22^	*U*^33^	*U*^12^	*U*^13^	*U*^23^
C2	0.0354 (16)	0.0361 (14)	0.0392 (16)	0.0024 (12)	0.0045 (13)	0.0010 (12)
C3	0.0363 (13)	0.0348 (14)	0.0400 (16)	0.0019 (12)	0.0034 (11)	0.0004 (12)
C4	0.0388 (16)	0.0376 (15)	0.0361 (16)	0.0038 (12)	0.0067 (13)	0.0045 (12)
C5	0.0343 (16)	0.0440 (16)	0.0405 (16)	0.0059 (13)	0.0016 (13)	0.0028 (13)
C6	0.0360 (16)	0.0441 (16)	0.0382 (16)	−0.0008 (13)	0.0024 (13)	0.0024 (13)
C7	0.0413 (18)	0.062 (2)	0.056 (2)	−0.0035 (15)	−0.0059 (15)	−0.0018 (16)
C8	0.0408 (18)	0.073 (2)	0.068 (2)	0.0067 (17)	−0.0063 (16)	−0.0006 (19)
C9	0.0442 (19)	0.060 (2)	0.064 (2)	0.0132 (16)	−0.0039 (16)	0.0023 (17)
C21	0.0464 (18)	0.0350 (15)	0.059 (2)	0.0044 (13)	0.0007 (15)	−0.0004 (14)
C22	0.0360 (16)	0.0403 (17)	0.066 (2)	−0.0037 (13)	0.0006 (15)	−0.0141 (15)
C23	0.065 (2)	0.055 (2)	0.116 (3)	0.0057 (19)	0.018 (2)	−0.021 (2)
C24	0.077 (3)	0.088 (3)	0.165 (6)	0.005 (3)	0.040 (4)	−0.054 (4)
C25	0.094 (4)	0.144 (5)	0.105 (4)	−0.022 (4)	0.039 (3)	−0.046 (4)
C26	0.090 (3)	0.148 (5)	0.076 (3)	−0.005 (3)	0.023 (3)	0.003 (3)
C27	0.067 (2)	0.088 (3)	0.070 (3)	0.009 (2)	0.013 (2)	0.008 (2)
C31	0.0387 (14)	0.0345 (15)	0.0511 (18)	0.0053 (12)	−0.0003 (13)	−0.0038 (13)
C41	0.0363 (16)	0.0360 (15)	0.0424 (17)	0.0040 (12)	0.0092 (13)	0.0014 (12)
C42	0.056 (2)	0.0439 (17)	0.0476 (19)	0.0069 (15)	0.0013 (15)	0.0040 (14)
C43	0.068 (2)	0.0391 (17)	0.060 (2)	0.0125 (16)	0.0060 (18)	0.0093 (15)
C44	0.059 (2)	0.0365 (16)	0.060 (2)	0.0046 (14)	0.0188 (17)	−0.0043 (15)
C45	0.061 (2)	0.0458 (18)	0.0416 (18)	−0.0007 (15)	0.0132 (15)	−0.0035 (14)
C46	0.0503 (18)	0.0386 (16)	0.0443 (19)	0.0050 (13)	0.0059 (15)	0.0052 (13)
N1	0.0380 (14)	0.0380 (13)	0.0447 (14)	0.0004 (11)	0.0003 (11)	−0.0014 (11)
N2	0.0420 (14)	0.0399 (14)	0.0596 (16)	0.0090 (11)	−0.0054 (12)	−0.0115 (12)
N3	0.0459 (17)	0.0522 (17)	0.086 (2)	0.0050 (13)	−0.0084 (16)	−0.0154 (15)
Br1	0.1319 (4)	0.0405 (2)	0.0945 (3)	0.0012 (2)	0.0293 (3)	−0.01699 (19)

### Geometric parameters (Å, º)

**Table:**

C2—N1	1.343 (3)	C22—C27	1.378 (5)
C2—N2	1.349 (3)	C23—C24	1.418 (7)
C2—C3	1.416 (4)	C23—H23	0.9300
C3—C4	1.403 (4)	C24—C25	1.363 (8)
C3—C31	1.424 (4)	C24—H24	0.9300
C4—C5	1.390 (4)	C25—C26	1.343 (7)
C4—C41	1.478 (4)	C25—H25	0.9300
C5—C6	1.381 (4)	C26—C27	1.372 (6)
C5—C9	1.508 (4)	C26—H26	0.9300
C6—N1	1.333 (4)	C27—H27	0.9300
C6—C7	1.500 (4)	C31—N3	1.139 (4)
C7—C8	1.519 (5)	C41—C42	1.388 (4)
C7—H7A	0.9700	C41—C46	1.391 (4)
C7—H7B	0.9700	C42—C43	1.375 (4)
C8—C9	1.531 (5)	C42—H42	0.9300
C8—H8A	0.9700	C43—C44	1.371 (5)
C8—H8B	0.9700	C43—H43	0.9300
C9—H9A	0.9700	C44—C45	1.371 (4)
C9—H9B	0.9700	C44—Br1	1.895 (3)
C21—N2	1.442 (3)	C45—C46	1.372 (4)
C21—C22	1.500 (4)	C45—H45	0.9300
C21—H21A	0.9700	C46—H46	0.9300
C21—H21B	0.9700	N2—H2	0.8600
C22—C23	1.368 (5)		
			
N1—C2—N2	118.3 (2)	C23—C22—C21	120.6 (3)
N1—C2—C3	121.3 (2)	C27—C22—C21	120.8 (3)
N2—C2—C3	120.3 (3)	C22—C23—C24	119.8 (4)
C4—C3—C2	121.3 (3)	C22—C23—H23	120.1
C4—C3—C31	121.4 (2)	C24—C23—H23	120.1
C2—C3—C31	117.3 (2)	C25—C24—C23	119.0 (4)
C5—C4—C3	115.9 (2)	C25—C24—H24	120.5
C5—C4—C41	123.8 (2)	C23—C24—H24	120.5
C3—C4—C41	120.3 (3)	C26—C25—C24	121.4 (5)
C6—C5—C4	119.0 (3)	C26—C25—H25	119.3
C6—C5—C9	110.1 (3)	C24—C25—H25	119.3
C4—C5—C9	130.9 (3)	C25—C26—C27	119.7 (5)
N1—C6—C5	126.1 (3)	C25—C26—H26	120.2
N1—C6—C7	122.6 (3)	C27—C26—H26	120.2
C5—C6—C7	111.3 (3)	C26—C27—C22	121.5 (4)
C6—C7—C8	103.1 (3)	C26—C27—H27	119.2
C6—C7—H7A	111.1	C22—C27—H27	119.2
C8—C7—H7A	111.1	N3—C31—C3	175.7 (3)
C6—C7—H7B	111.1	C42—C41—C46	117.7 (3)
C8—C7—H7B	111.1	C42—C41—C4	121.0 (3)
H7A—C7—H7B	109.1	C46—C41—C4	121.3 (2)
C7—C8—C9	106.5 (3)	C43—C42—C41	121.8 (3)
C7—C8—H8A	110.4	C43—C42—H42	119.1
C9—C8—H8A	110.4	C41—C42—H42	119.1
C7—C8—H8B	110.4	C44—C43—C42	118.6 (3)
C9—C8—H8B	110.4	C44—C43—H43	120.7
H8A—C8—H8B	108.6	C42—C43—H43	120.7
C5—C9—C8	103.1 (3)	C45—C44—C43	121.5 (3)
C5—C9—H9A	111.2	C45—C44—Br1	119.1 (3)
C8—C9—H9A	111.1	C43—C44—Br1	119.4 (2)
C5—C9—H9B	111.1	C44—C45—C46	119.3 (3)
C8—C9—H9B	111.2	C44—C45—H45	120.3
H9A—C9—H9B	109.1	C46—C45—H45	120.3
N2—C21—C22	113.8 (2)	C45—C46—C41	121.1 (3)
N2—C21—H21A	108.8	C45—C46—H46	119.4
C22—C21—H21A	108.8	C41—C46—H46	119.4
N2—C21—H21B	108.8	C6—N1—C2	116.4 (2)
C22—C21—H21B	108.8	C2—N2—C21	125.7 (2)
H21A—C21—H21B	107.7	C2—N2—H2	117.2
C23—C22—C27	118.6 (4)	C21—N2—H2	117.2
			
N1—C2—C3—C4	2.1 (4)	C23—C24—C25—C26	0.2 (8)
N2—C2—C3—C4	−178.0 (3)	C24—C25—C26—C27	−1.0 (9)
N1—C2—C3—C31	−174.6 (3)	C25—C26—C27—C22	0.2 (7)
N2—C2—C3—C31	5.3 (4)	C23—C22—C27—C26	1.4 (6)
C2—C3—C4—C5	−0.6 (4)	C21—C22—C27—C26	−177.3 (4)
C31—C3—C4—C5	175.9 (3)	C5—C4—C41—C42	−47.5 (4)
C2—C3—C4—C41	−179.5 (2)	C3—C4—C41—C42	131.3 (3)
C31—C3—C4—C41	−3.0 (4)	C5—C4—C41—C46	134.4 (3)
C3—C4—C5—C6	−0.5 (4)	C3—C4—C41—C46	−46.8 (4)
C41—C4—C5—C6	178.3 (3)	C46—C41—C42—C43	1.0 (4)
C3—C4—C5—C9	−179.1 (3)	C4—C41—C42—C43	−177.1 (3)
C41—C4—C5—C9	−0.3 (5)	C41—C42—C43—C44	−1.3 (5)
C4—C5—C6—N1	0.3 (4)	C42—C43—C44—C45	0.8 (5)
C9—C5—C6—N1	179.1 (3)	C42—C43—C44—Br1	−178.9 (2)
C4—C5—C6—C7	−178.9 (3)	C43—C44—C45—C46	−0.1 (5)
C9—C5—C6—C7	0.0 (4)	Br1—C44—C45—C46	179.7 (2)
N1—C6—C7—C8	166.1 (3)	C44—C45—C46—C41	−0.2 (5)
C5—C6—C7—C8	−14.7 (4)	C42—C41—C46—C45	−0.2 (4)
C6—C7—C8—C9	23.4 (4)	C4—C41—C46—C45	177.9 (3)
C6—C5—C9—C8	14.7 (3)	C5—C6—N1—C2	1.1 (4)
C4—C5—C9—C8	−166.6 (3)	C7—C6—N1—C2	−179.8 (3)
C7—C8—C9—C5	−23.4 (4)	N2—C2—N1—C6	177.9 (3)
N2—C21—C22—C23	138.8 (3)	C3—C2—N1—C6	−2.2 (4)
N2—C21—C22—C27	−42.5 (4)	N1—C2—N2—C21	3.3 (4)
C27—C22—C23—C24	−2.2 (5)	C3—C2—N2—C21	−176.6 (3)
C21—C22—C23—C24	176.6 (3)	C22—C21—N2—C2	98.5 (3)
C22—C23—C24—C25	1.4 (7)		

### Hydrogen-bond geometry (Å, º)

**Table:**

*D*—H···*A*	*D*—H	H···*A*	*D*···*A*	*D*—H···*A*
N2—H2···N3^i^	0.86	2.23	2.974 (4)	145

Symmetry code: (i) −*x*+1, −*y*, −*z*+1.
